# MicroRNA-7450 regulates non-thermal plasma-induced chicken Sertoli cell apoptosis via adenosine monophosphate-activated protein kinase activation

**DOI:** 10.1038/s41598-018-27123-8

**Published:** 2018-06-08

**Authors:** Jiao Jiao Zhang, Xian Zhong Wang, Huynh Luong Do, Nisansala Chandimali, Tae Yoon Kang, Nameun Kim, Mrinmoy Ghosh, Sang Baek Lee, Young Sun Mok, Seong Bong Kim, Taeho Kwon, Dong Kee Jeong

**Affiliations:** 1 Laboratory of Animal Genetic Engineering and Stem Cell Biology, Department of Advanced Convergence Technology and Science, Jeju National University, Jeju, 63243 Republic of Korea; 2 Chongqing Key Laboratory of Forage and Herbivore, College of Animal Science and Technology, Southwest University, Chongqing, 400715 P. R. China; 3 Department of Chemical and Biological Engineering, Jeju National University, Jeju, 63243 Republic of Korea; 4 Plasma Technology Research Center, National Fusion Research Institute, Gunsan-si, Jeollabuk-Do, 54004 Republic of Korea; 5 Laboratory of Animal Genetic Engineering and Stem Cell Biology, Subtropical/Tropical Organism Gene Bank, Jeju National University, Jeju, 63243 Republic of Korea

## Abstract

Non-thermal plasma treatment is an emerging innovative technique with a wide range of biological applications. This study was conducted to investigate the effect of a non-thermal dielectric barrier discharge plasma technique on immature chicken Sertoli cell (SC) viability and the regulatory role of microRNA (miR)-7450. Results showed that plasma treatment increased SC apoptosis in a time- and dose-dependent manner. Plasma-induced SC apoptosis possibly resulted from the excess production of reactive oxygen species via the suppression of antioxidant defense systems and decreased cellular energy metabolism through the inhibition of adenosine triphosphate (ATP) release and respiratory enzyme activity in the mitochondria. In addition, plasma treatment downregulated miR-7450 expression and activated adenosine monophosphate-activated protein kinase α (AMPKα), which further inhibited mammalian target of rapamycin (mTOR) phosphorylation in SCs. A single-stranded synthetic miR-7450 antagomir disrupted mitochondrial membrane potential and decreased ATP level and mTOR phosphorylation by targeting the activation of AMPKα, which resulted in significant increases in SC lethality. A double-stranded synthetic miR-7450 agomir produced opposite effects on these parameters and ameliorated plasma-mediated apoptotic effects on SCs. Our findings suggest that miR-7450 is involved in the regulation of plasma-induced SC apoptosis through the activation of AMPKα and the further inhibition of mTOR signaling pathway.

## Introduction

The use of non-thermal plasma is receiving great interest in various biomedical applications, including sterilization, blood coagulation, wound healing, tissue regeneration, dental treatment, promotion of cell transfection efficiency, cell proliferation and differentiation, and cancer therapy^1,2^. Numerous plasma sources are being commercialized for medical use, such as volume dielectric barrier discharges (DBDs), atmospheric pressure plasma jets, coronas, and surface and microwave discharges, which need to be extensively optimized to ensure their safe application on living cells or tissues^1,3^. Our laboratory has established a non-thermal DBD plasma system generated in argon at atmospheric pressure by applying a high voltage between a dielectric-covered electrode and the biological target, which creates electrically safe plasma^4–6^. Our previous studies have suggested that appropriate non-thermal DBD plasma treatment conditions need to be optimized for the development of chicken embryo during the early stages of incubation^5^ and for the improvement in chicken growth and male reproductive capacity, particularly sperm quality^6^. We have a hypothesis that the plasma treatment may affect SCs in prepubertal chickens, which were more proliferative than those in pubertal chickens^7^ and play an important role in regulating spermatogenesis and supporting germ cell development^8^. The present study was carried out with the objectives to investigate the effect of non-thermal plasma treatment on immature chicken Sertoli cell (SC) viability and growth *in vitro* and the exploration of plasma exposure condition before its *in vivo* application.

SCs play an important physiological role in the testes, where they support, nourish, and protect germ cells and are required for the appropriate differentiation of germ cells^8^. Each SC provides support to a limited number of differentiating germ cells in the seminiferous tubule and provides them with growth factors, binding proteins, and energy in the form of lactate, thereby promoting germ cell growth and differentiation into spermatozoa; therefore, the number of SCs is important for spermatozoa production^9^. The proliferation of immature SCs affects the final number of mature SCs, which in turn determines testicular size and spermatogenesis competence in the male reproductive system^10^. SCs are highly sensitive to internal signals such as systemic energy levels, growth factors, and hormones^11,12^ and exhibit increased metabolism that allows them to support germ cells^13^. Strikingly, mammalian SCs are relatively resistant to apoptosis in response to DNA damage. It has been shown that SCs easily survive high doses of radiation exposure in developing rat testes^14^ and that a mild apoptotic SC response is observed following exposure to ionizing radiation in human fetuses^15^. Thus, the exposure condition of plasma should be sufficiently optimized when it is applied on SCs, based on the facts that dose-dependent effects of plasma on many types of normal and cancer cells (including fibroblasts^16^, endothelial cells^17^, epithelial cells^18^, myoblasts^19^, keratinocytes^20^, and various tumor cells^21^, etc.).

The controlled delivery of reactive species (atoms, radicals, ions, electrons, ultraviolet photons, and reactive oxygen and nitrogen species, etc.) produced in non-thermal atmospheric pressure plasma to the surface and interior of plasma-treated living cells or tissues results in biological responses such as altered metabolism and programmed cell death^22,23^. Abundant evidence from recent studies has shown that plasma-generated reactive oxygen species (ROS) effectively induce biological effects that vary from increased cell proliferation^17^ to cell apoptosis in many types of normal cells^24,25^ and cancer cells^26^. There are numerous possibilities regarding how plasma influences cells at a molecular^27^ and genetic^28^ level. However, knowledge of cellular signaling events following plasma treatment of eukaryotic cells or tissues is still rudimentary. In the present study, we investigated the molecular and cellular mechanisms of non-thermal DBD plasma-induced *in vitro* effects on chicken SCs, with particular emphasis on the roles of intracellular ROS and energy metabolism in regulating gene expression and cellular signaling pathways, which may provide a potential reference for the vivo application in future.

MicroRNAs (miRNAs) control a wide range of biological processes, including cell proliferation, migration, differentiation, apoptosis, and metabolism, by mediating RNA silencing and regulating gene expression at the post-transcriptional level^29,30^. Recent studies have demonstrated that miRNAs repress the expression of SC target genes, thereby controlling SC physiology in functions such as proliferation, maturation, apoptosis, formation of tight junctions and the blood–testis barrier, and hormone responses in the seminiferous epithelium cycle^12,31^. Ablation of SC dicer, which is an enzyme essential for SC maturation and survival and is ultimately required to sustain germ cell development, leads to infertility. This observation indicates that miRNA expression in SCs is important in supporting germ cell development^32^. Our previous work with miRNA arrays found that overdoses of non-thermal plasma treatment in immature chicken SCs cultured *in vitro* downregulate miR-7450, whose predicted target gene is adenosine monophosphate-activated protein kinase α (AMPKα; unpublished data). AMPK is a key sensor and regulator of cellular energy homeostasis. Activated AMPK inhibits cell growth and induces cell apoptosis via the suppression of mammalian target of rapamycin (mTOR) signaling pathway^33–35^, which is regulated by the lysosomal permeabilization, protein synthesis and transcription, and activated phosphorylation on serine 2448^36^. In SCs, the AMPK-mTOR signaling pathway has been found to be involved in the disruption of polarity^37^, autophagy, and programmed cell death induced by toxic substances^38,39^. The present study aimed to elucidate the role of miR-7450 in the regulation of non-thermal plasma-mediated effects on SCs through the AMPK-mTOR signaling pathway.

## Results

### Effect of non-thermal plasma on chicken SC viability and apoptosis

**Figure 1:**
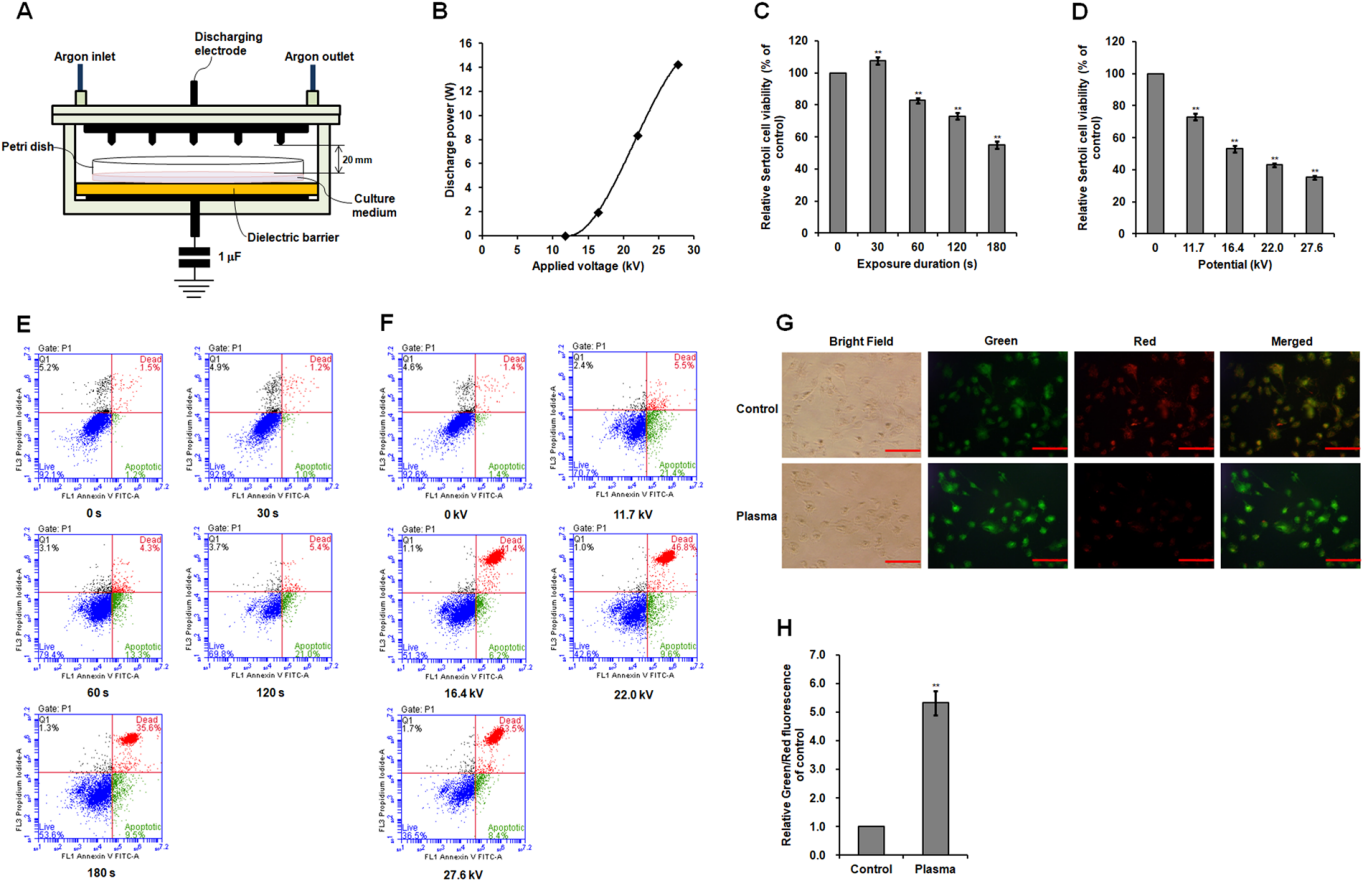
Chicken SC viability and apoptosis after non-thermal plasma treatment. (**A**) Schematic of non-thermal DBD plasma reactor. (**B**) Dependence of the discharge power on the applied voltage. (**C**) Relative viability in SCs exposed to 11.7 kV for different durations. (**D**) Relative viability in SCs exposed to different potentials for 120 s. (**E**) Flow cytometric analysis of cell apoptosis in SCs exposed to 11.7 kV for different durations. (**F**) Flow cytometric analysis of cell apoptosis in SCs exposed to different potentials for 120 s. (**G**) JC-1 staining of SCs exposed to 22.0 kV of plasma for 120 s. Scale bar: 50 μm. (**H**) Relative green/red fluorescence intensity for JC-1 staining. Data are represented as the mean ± SD (n = 3 per group). *p < 0.05; **p < 0.01, according to one-way ANOVA and LSD test.

Exposure to 11.7 kV of non-thermal plasma for 30 s increased SC viability and growth (Fig. 1C; Supplementary Fig. S1), while prolonged plasma exposure of more than 60 s exerted inhibitory effects on cell viability and significant cell apoptosis (Fig. 1C,E; Supplementary Fig. S1). SC viability and growth decreased after exposure to varying plasma potentials (11.7–27.6 kV) for 120 s (Fig. 1D; Supplementary Fig. S2). These plasma treatments induced significant increases in the number of dead SCs (Fig. 1F), and non-thermal plasma inflicted both time- and dose-dependent damage on chicken SCs cultured *in vitro*. Considering less than half percentages of cell viability, we used an intensity of 22.0 kV and exposure duration of 120 s for all further mechanistic analyses of plasma-induced apoptotic effects on SCs.

Green fluorescence of JC-1 dye indicates a decrease in mitochondrial membrane potential, which is observed early in cell apoptosis, and mitochondrial depolarization occurring during apoptosis is indicated by an increase in the green/red fluorescence intensity ratio. SCs exposed to 22.0 kV of non-thermal plasma for 120 s displayed significantly increased JC-1 green fluorescence intensity (Fig. 1G) and exhibited a 4.32-fold increase in the green/red fluorescence intensity ratio compared to that in the control group (*p* < 0.001; Fig. 1H), this indicated that plasma treatment significantly decreased the mitochondrial membrane potential of SCs and increased the mitochondrial depolarization occurring in cell apoptosis.

### Effect of non-thermal plasma on ROS production and antioxidants

**Figure 2:**
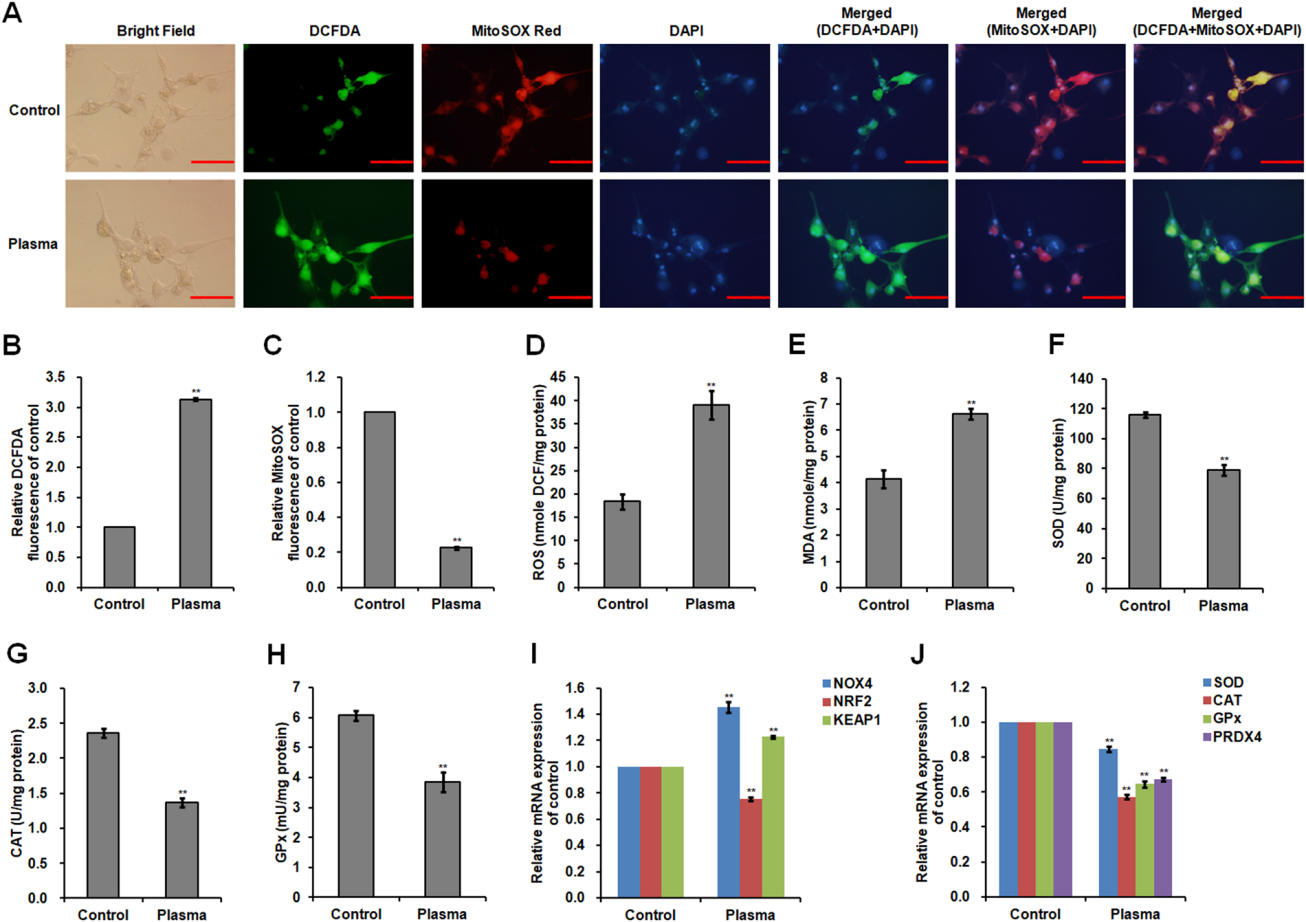
ROS production and antioxidant activity of plasma-treated SCs. Chicken SCs were exposed to 22.0 kV of non-thermal plasma for 120 s. (**A**) Imaging of SCs stained with DCFDA/MitoSOX Red/DAPI. Intracellular ROS production was detected by DCFDA staining and mitochondrial superoxide was detected by MitoSOX Red staining. DCFDA: green fluorescence; MitoSOX Red: red fluorescence; DAPI: blue fluorescence. Scale bar: 50 μm. (**B**) Relative fluorescence intensity for DCFDA staining. (**C**) Relative fluorescence intensity for MitoSOX Red staining. (**D**) Total ROS levels in SCs. (**E**) MDA level in SCs. Activities of (**F**) SOD, (**G**) CAT, and (**H**) GPx in SCs. Relative mRNA levels of (**I**) *NOX4*, *NRF2*, and *KEAP1*, and (**J**) *SOD*, *CAT*, *GPx*, and *PRDX4*. Data are represented as the mean ± SD (n = 3 per group). *p < 0.05; **p < 0.01, according to one-way ANOVA and LSD test.

**Figure 3:**
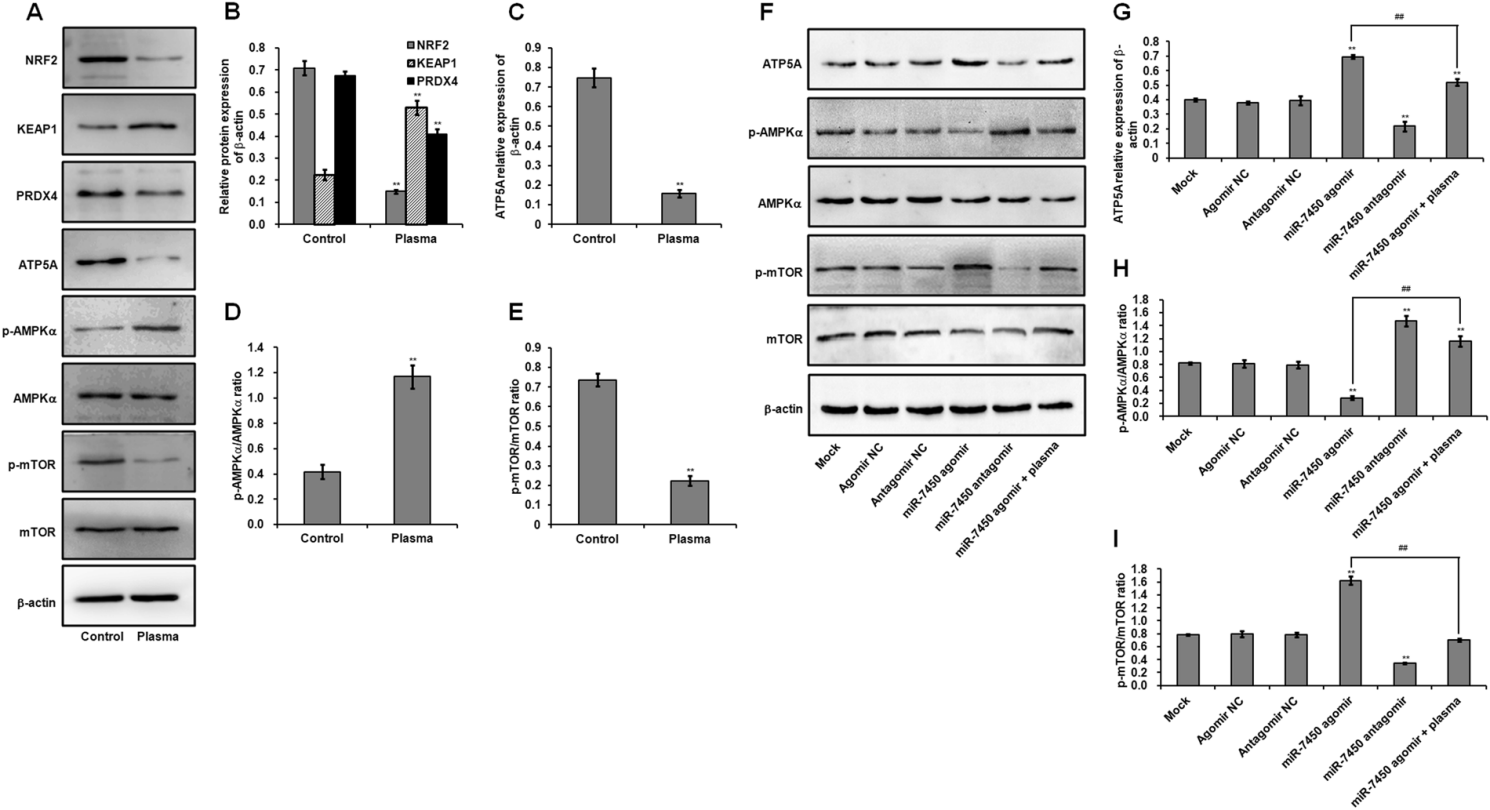
Chicken SC protein expression. (**A**) Representative western blot analysis of protein bands in SCs exposed to 22.0 kV of plasma for 120 s. Uncropped immunoblot scans are presented in Supplementary Figure S5. Relative protein levels of (**B**) NRF2, KEAP1, PRDX4, (**C**) ATP5A, (**D**) p-AMPKα/AMPKα, and (**E**) p-mTOR/mTOR in SCs exposed to plasma. (**F**) Representative western blot analysis of protein bands in SCs trasfected with miR-7450 agomir and antagomir, and miR-7450 agomir-transfected group treated with 22.0 kV of plasma for 120 s. Uncropped immunoblot scans are presented in Supplementary Figure S6. Relative protein levels of (**G**) ATP5A, (**H**) p-AMPKα/AMPKα, and (**I**) p-mTOR/mTOR in transfected SCs. One independent replicate on western blot analysis of protein bands in SCs is presented in Supplementary Figure S7. Data are represented as the mean ± SD (n = 3 per group). *p < 0.05; **p < 0.01; ^##^*p* < 0.01, according to one-way ANOVA and LSD test.

Compared to the control group, plasma-treated SCs increased 2′,7′-dichlorodihydrofluorescein diacetate (DCFDA) fluorescence intensity (which reflects the amount of intracellular ROS generation) 3.13-fold (*p* < 0.001; Fig. 2A,B) but decreased 0.77-fold (*p* < 0.001) in MitoSOX fluorescence intensity (indicating superoxide levels generated in the mitochondria of live SCs; Fig. 2A,C). The total ROS and malondialdehyde (MDA) levels in plasma-treated SCs increased 1.12-fold (*p* = 0.001; Fig. 2D) and 0.60-fold (*p* = 0.001; Fig. 2E), respectively. mRNA expression of nicotinamide adenine dinucleotide phosphate oxidase 4 (*NOX4*), which is an enzyme that produces ROS, increased following plasma treatment (*p* < 0.001; Fig. 2I), while nuclear factor erythroid 2-related factor 2 (*NRF2*) mRNA expression (*p* < 0.001; Fig. 2I) and protein (*p* < 0.001; Fig. 3A,B) level decreased. NRF2 regulates the expression of antioxidant proteins that protect against oxidative damage. Furthermore, plasma treatment induced increases of 0.23-fold (*p* < 0.001; Fig. 2I) and 1.36-fold (*p* < 0.001; Fig. 3A,B) in mRNA and protein expression of kelch-like ECH-associated protein 1 (KEAP1), which interacts with NRF2 and facilitates its degradation in the cytoplasm.

The antioxidant enzymes superoxide dismutase (SOD), catalase (CAT), and glutathione peroxidase (GPx) in plasma-treated SCs exhibited decreases of 0.32- (*p* < 0.001; Fig. 2F), 0.42- (p < 0.001; Fig. 2G), and 0.37-fold (*p* = 0.001; Fig. 2H), respectively; *SOD*, *CAT*, and *GPx* mRNA levels also decreased (*p* < 0.001; Fig. 2J). Non-thermal plasma treatment also led to a 0.33-fold decrease in SC peroxiredoxin 4 (*PRDX4*) gene expression (*p* < 0.001; Fig. 2J) and a 0.40-fold decrease in PRDX4 protein expression (*p* < 0.001; Fig. 3A,B).

### Effect of non-thermal plasma on mitochondria activity, mitochondrial respiratory enzyme, and ATP level

**Figure 4:**
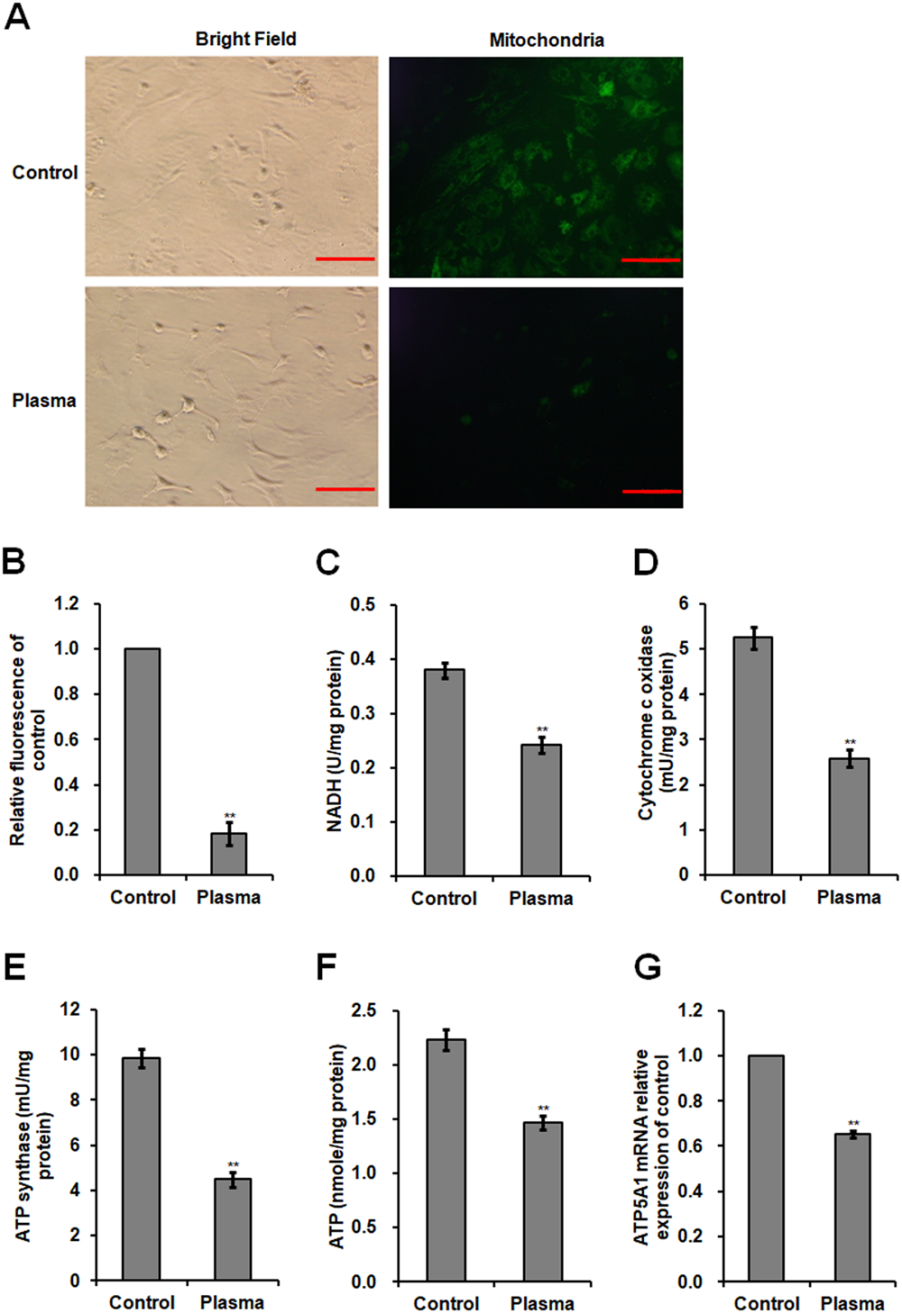
Mitochondria activity, mitochondrial respiratory enzyme, and ATP level of plasma-treated SCs. Chicken SCs were exposed to 22.0 kV of non-thermal plasma for 120 s. (**A**) Imaging of SCs stained with a Cell Navigator Mitochondrial Staining Kit (green fluorescence). Scale bar: 50 μm. (**B**) Relative fluorescence intensity for mitochondrial staining. (**C**) NADH level. Activities of (**D**) cytochrome c oxidase and (**E**) ATPase synthase in the mitochondria of SCs. (**F**) ATP level in SCs. (**G**) *ATP5A1* mRNA relative level. Data are represented as the mean ± SD (n = 3 per group). *p < 0.05; **p < 0.01, according to one-way ANOVA and LSD test.

Mitochondrial fluorescence intensity in plasma-treated SCs decreased 0.82-fold (*p* < 0.001; Fig. 4A,B). NADH level and enzymatic activities of cytochrome c oxidase and ATP synthase decreased 0.36- (*p* = 0.001; Fig. 4C), 0.51- (p < 0.001; Fig. 4D), and 0.54-fold (*p* < 0.001; Fig. 4E), respectively. Plasma treatment exhibited a 0.34-fold decrease in ATP level (*p* = 0.001; Fig. 4F), a 0.35-fold decrease in *ATP5A1* mRNA level (*p* < 0.001; Fig. 4G), and a 0.79-fold decrease in ATP5A protein expression (*p* < 0.001; Fig. 3A,C).

### Effect of non-thermal plasma on miR-7450 level and gene and protein expression of AMPKα and mTOR

**Figure 5:**
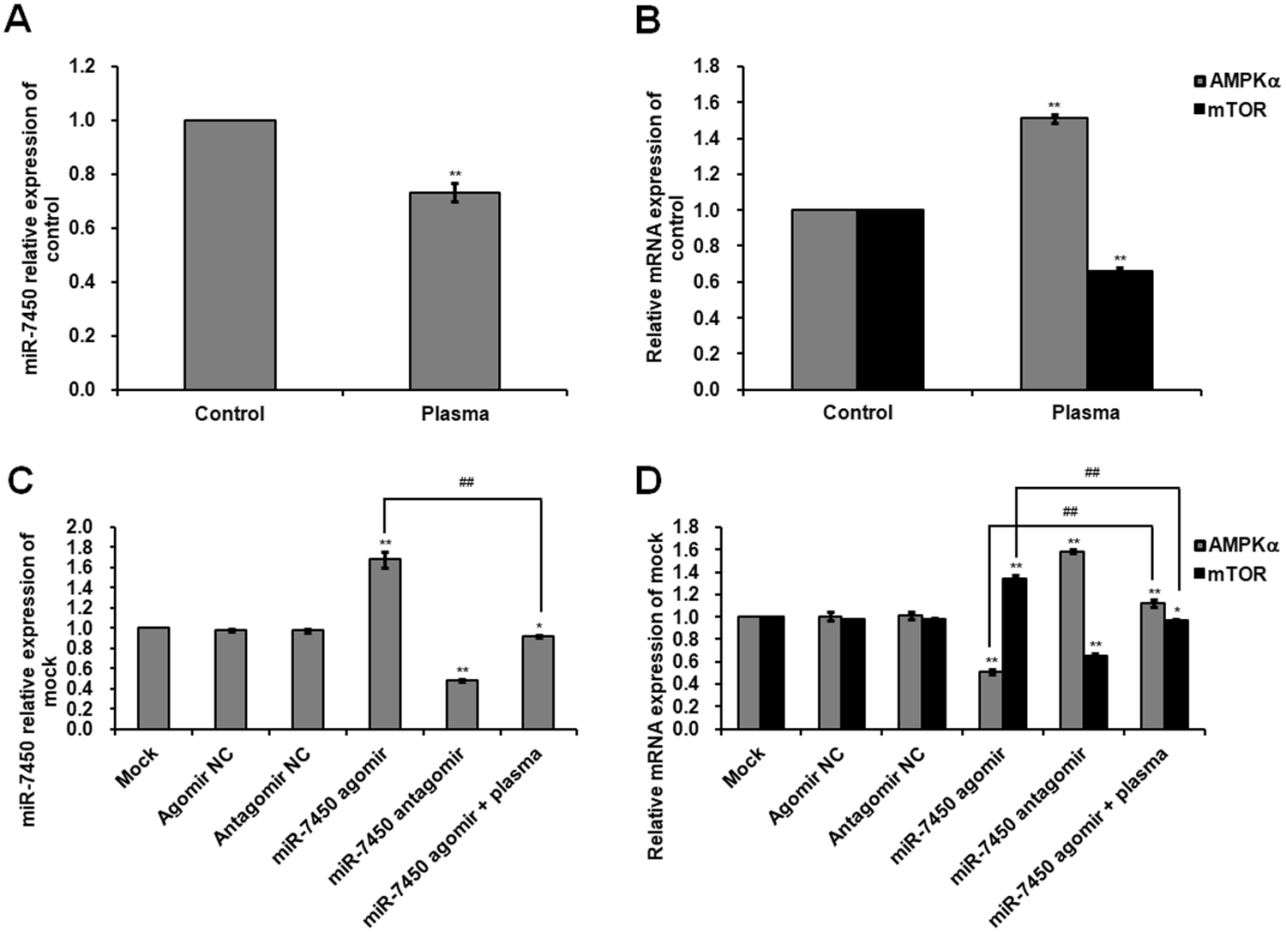
Expression of miR-7450 and mRNA levels of *AMPKα* and *mTOR* in SCs. (**A**) miR-7450 relative level and (**B**) relative mRNA levels of *AMPKα* and *mTOR* in SCs exposed to 22.0 kV of plasma for 120 s. (**C**) miR-7450 relative level and (**D**) relative mRNA levels of *AMPKα* and *mTOR* in SCs transfected with miR-7450 agomir and antagomir, and miR-7450 agomir-transfected group treated with 22.0 kV of plasma for 120 s. RT-PCR analysis of a non-target gene (*POU1F1*) and an unrelated target gene (*PDE10A*) of miR-7450 in transfected SCs is presented in Supplementary Figure S4. Data are represented as the mean ± SD (n = 3 per group). *p < 0.05; **p < 0.01; ^##^*p* < 0.01, according to one-way ANOVA and LSD test.

Our previous work using miRNA arrays found that overdoses of non-thermal plasma treatment downregulated miR-7450 level in chicken SCs cultured *in vitro*. Here we performed RT-PCR analysis of miR-7450 and the expression of its target gene (*AMPKα*, which was predicted by miRDB; http://www.mirdb.org/) and a downstream kinase mTOR. Plasma treatment induced a 0.27-fold decrease in miR-7450 level (*p* < 0.001; Fig. 5A), but a 0.51-fold increase in *AMPKα* mRNA expression (*p* < 0.001; Fig. 5B) and a 1.80-fold increase in AMPKα phosphorylation (*p* < 0.001; Fig. 3A,D) compared to those of the control group. Plasma treatment also exhibited decreases of 0.34- and 0.70-fold in *mTOR* mRNA expression and mTOR phosphorylation (*p* < 0.001; Figs 3A,E, 5B), respectively. However, significantly differential mRNA expression of *AMPKα* and *mTOR* in SCs after plasma treatment was not reflected on corresponding protein expression in western blot analyses (Figs 3A, 5B), this discrepancy can be explained by the facts that transcription, mRNA decay, translation, and protein degradation are key processes determining steady state protein abundance, which result in the unnecessary correlation between mRNA levels and corresponding protein expression^40–44^.

### Effect of miR-7450 agomir and antagomir on SC viability and apoptosis

**Figure 6:**
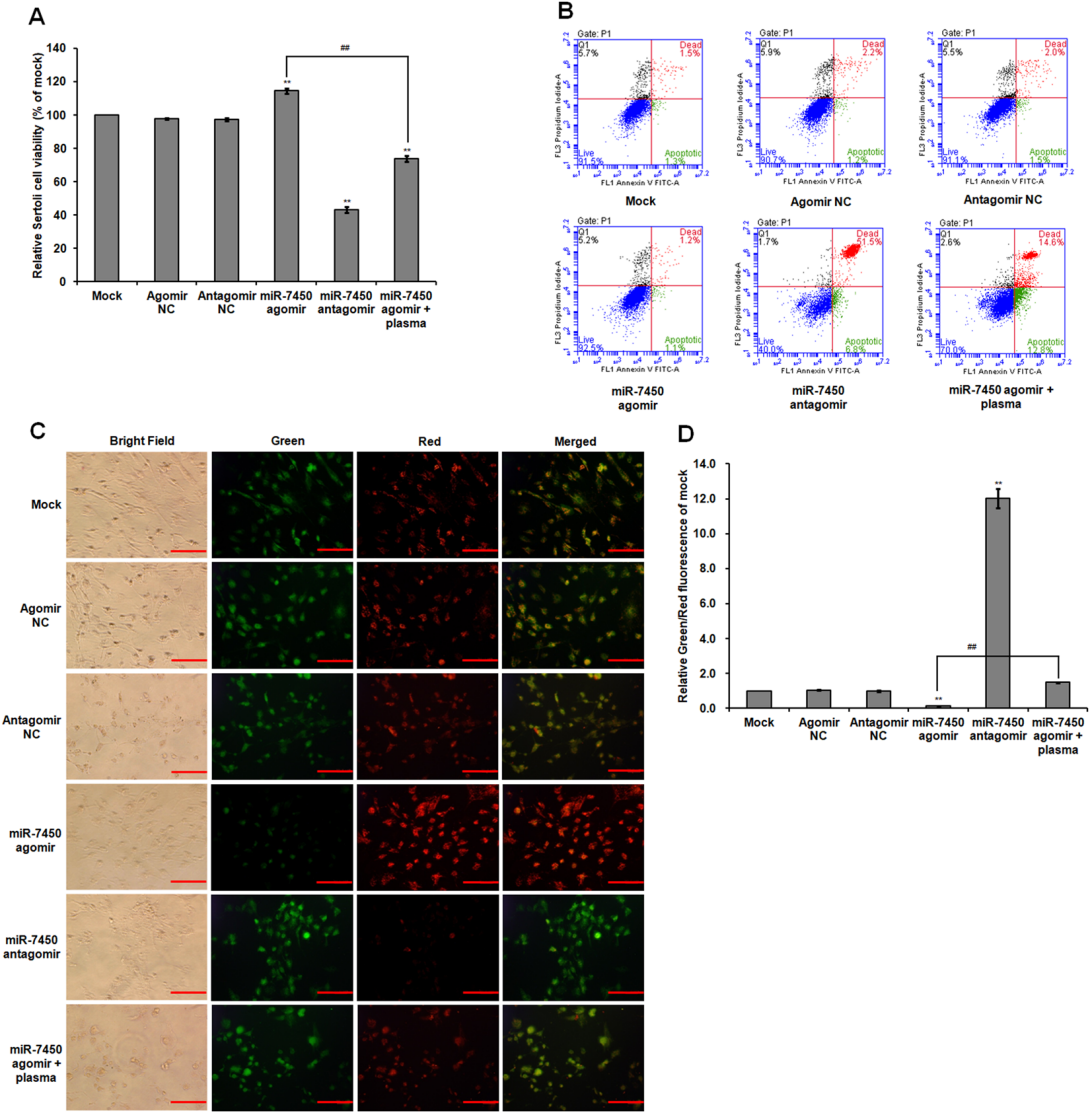
SC viability and apoptosis after miRNA transfection. Chicken SCs were transfected with miR-7450 agomir and antagomir, and miR-7450 agomir-transfected group treated with 22.0 kV of plasma for 120 s. (**A**) Relative viability of SCs. (**B**) Flow cytometric analysis of SC cell apoptosis. (**C**) JC-1 staining of SCs. Scale bar: 50 μm. (**D**) Relative green/red fluorescence intensity for JC-1 staining. Data are represented as the mean ± SD (n = 3 per group). *p < 0.05; **p < 0.01; ^##^*p* < 0.01, according to one-way ANOVA and LSD test.

Neither negative control produced statistically significant effects when compared with the mock group (*p >* 0.05; Fig. 6). Compared to the mock group, the miR-7450 antagomir (a single-stranded synthetic miR-7450 inhibitor) significantly inhibited SC viability and growth in transfected cells, but the miR-7450 agomir (a double-stranded synthetic miR-7450 mimic) showed a 0.15-fold increase in SC viability (*p* < 0.001; Fig. 6A; Supplementary Fig. S3). Moreover, the miR-7450 agomir weakened the inhibitory effect of non-thermal plasma treatment on SC viability, with a 0.35-fold decrease when compared to that in the miR-7450 agomir-transfected group (*p* < 0.001; Fig. 6A). Compared with the mock group, miR-7450 antagomir significantly increased apoptotic and dead SCs (58.3%), but the miR-7450 agomir did not show any significant differences (Fig. 6B), which indicated that miR-7450 agomir had no damage effect on SC growth. In addition, non-thermal plasma treatment at 22.0 kV for 120 s exhibited 56.4% apoptotic and dead cells (Fig. 1F), but miR-7450 agomir reduced cell apoptosis induced by plasma treatment, showing 27.4% apoptotic and dead cells (Fig. 6B).

The miR-7450 antagomir significantly increased JC-1 green fluorescence intensity (Fig. 6C), which indicates a decrease in mitochondrial membrane potential of SCs, and produced a 11.02-fold increase in the green/red fluorescence intensity ratio, which shows an increase in mitochondrial depolarization occurring in cell apoptosis, compared to that in the mock group (*p* < 0.001; Fig. 6D). Conversely, the miR-7450 agomir increased JC-1 red fluorescence intensity (Fig. 6C), resulting in a 0.87-fold decrease in the green/red fluorescence intensity ratio (*p* = 0.002; Fig. 6D), this indicated that miR-7450 agomir transfected SCs had higher mitochondrial membrane potential and percentage of mitochondria within a population than the mock-transfected (Lipofectamine^®^ RNAiMAX Regent only) group. In addition, miR-7450 agomir significantly weakened the inhibitory effect of non-thermal plasma treatment on mitochondrial membrane potential of SCs, showing increased JC-1 green fluorescence intensity (Fig. 6C) and a 10.50-fold increase in the green/red fluorescence intensity ratio (*p* < 0.001; Fig. 6D) when compared to that in the miR-7450 agomir-transfected group.

### Effect of miR-7450 agomir and antagomir on mitochondria activity, mitochondrial respiratory enzyme, and ATP level

**Figure 7:**
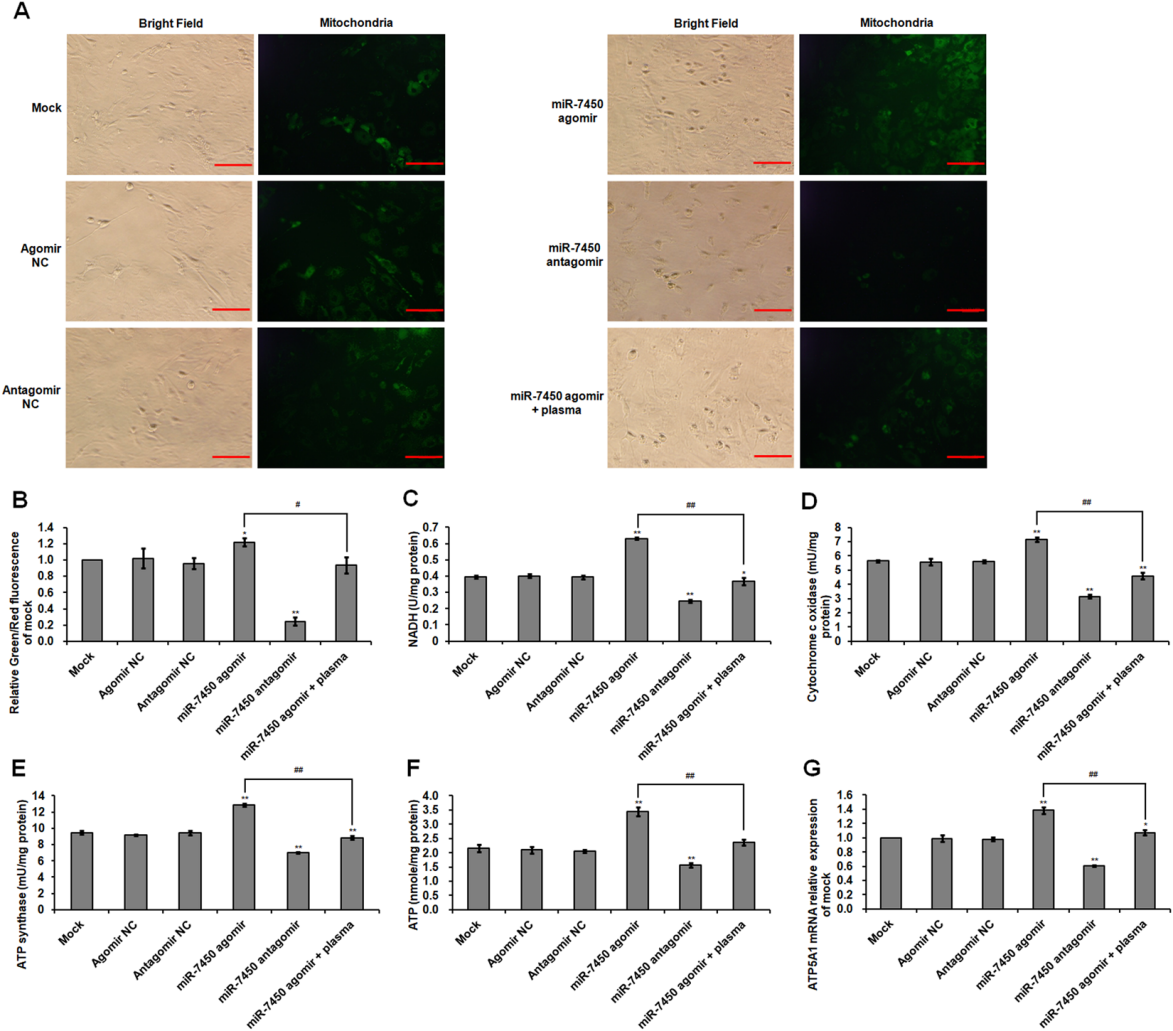
Mitochondria activity, mitochondrial respiratory enzyme, and ATP level of transfected SCs. Chicken SCs were transfected with miR-7450 agomir and antagomir, and miR-7450 agomir-transfected group treated with 22.0 kV of plasma for 120 s. (**A**) Mitochondrial staining in SCs. Scale bar: 50 μm. (**B**) Relative fluorescence intensity for mitochondrial staining. (**C**) NADH level. Activities of (**D**) cytochrome c oxidase and (**E**) ATPase synthase in the mitochondria of SCs. (**F**) ATP level in SCs. (**G**) *ATP5A1* mRNA relative level. Data are represented as the mean ± SD (n = 3 per group). *p < 0.05; **p < 0.01; ^#^*p* < 0.05; ^##^*p* < 0.01, according to one-way ANOVA and LSD test.

The miR-7450 antagomir induced a 0.75-fold (*p* < 0.001) decrease in mitochondrial fluorescence intensity of transfected SCs, while the miR-7450 agomir significantly increased fluorescence intensity and weakened the inhibitory effect of non-thermal plasma treatment (Fig. 7A,B). Compared to the mock group, NADH level and activities of cytochrome c oxidase and ATP synthase decreased 0.38- (*p* < 0.001; Fig. 7C), 0.44- (p < 0.001; Fig. 7D), and 0.26-fold (*p* < 0.001; Fig. 7E), respectively, following miR-7450 antagomir transfection. However, the miR-7450 agomir produced significant improvements in mitochondrial respiratory enzyme activity and ameliorated the inhibitory effect of non-thermal plasma treatment (Fig. 7C–E). In addition, ATP concentration, *ATP5A1* mRNA expression, and ATP5A protein level in SCs transfected with the miR-7450 antagomir decreased 0.27-fold (*p* < 0.001; Fig. 7F), 0.39-fold (*p* < 0.001; Fig. 7G), and 0.45-fold (*p* < 0.001; Fig. 3F,G), respectively, while the miR-7450 agomir significantly increased ATP production and *ATP5A1* mRNA and protein expression and reduced the inhibitory effect of non-thermal plasma treatment on ATP (Figs 3F,G, 7F,G).

### Effect of miR-7450 agomir and antagomir on miRNA level and gene and protein expression of AMPKα and mTOR

Compared to the mock group, the miR-7450 antagomir induced a 0.52-fold decrease in miR-7450 level (*p* < 0.001; Fig. 5C) and a 0.58-fold increase in *AMPKα* mRNA expression (*p* < 0.001; Fig. 5D) and a 0.80-fold increase in AMPKα phosphorylation (*p* < 0.001; Fig. 3F and H) and also downregulated *mTOR* mRNA expression and mTOR phosphorylation (*p* < 0.001; Figs 3F,I, 5D). Conversely, the miR-7450 agomir exhibited a 0.68-fold increase in miR-7450 level (*p* < 0.001; Fig. 5C), a 0.34-fold increase in *mTOR* mRNA expression (*p* < 0.001; Fig. 5D), and a 1.07-fold increase in mTOR phosphorylation (*p* < 0.001; Fig. 3F,I), but decreased *AMPKα* mRNA expression and AMPKα phosphorylation (*p* < 0.001; Figs 3F,H, 5D). In addition, the miR-7450 agomir significantly reduced the effects of non-thermal plasma treatment on miR-7450 level and mRNA expression and protein phosphorylation of AMPKα and mTOR, showing a 0.45-fold decrease in miR-7450 level (*p* < 0.001; Fig. 5C) and decreases of 0.28- and 0.57-fold in *mTOR* mRNA expression and mTOR phosphorylation (*p* < 0.001; Fig. 3F,I, 5D), respectively, but increases of 1.20- and 3.10-fold in *AMPKα* mRNA expression and AMPKα phosphorylation (*p* < 0.001; Fig. 3F,H, 5D), respectively.

Beside the facts that transcription, mRNA decay, translation, and protein degradation can control steady state protein levels^40–43^, a multitude of post-translational modifications also control protein turnover and abundance. Among those post-translational mechanisms, miRNAs are known to simultaneously repress hundreds of genes by inhibiting mRNA translation into protein^45^. Although the results showed significantly differential mRNA expression of *AMPKα* and *mTOR* in SCs after miR-7450 agomir and antagomir transfection was not reflected on corresponding protein expression in western blot analyses (Figs 3F, 5D), but significant changes of AMPKα and mTOR phosphorylation level were found. Post-translational phosphorylation status modulates the mechanisms which control cell function and affect protein levels^46^.

In order to show the specificity of miR-7450 on target genes, mRNA expression of POU class 1 homeobox 1 (*POU1F1*), which is not a target gene of miR-7450, was analyzed in transfected SCs. The result showed that the miR-7450 agomir and antagomir had no significant effect on *POU1F1* mRNA expression (Supplementary Figure S4). In order to exclude the possible systematic bias in SCs transfected with agomir and antagomir, we analyzed the mRNA expression of an unrelated target gene of miR-7450 (phosphodiesterase 10 A, *PDE10A*; Supplementary Figure S4). The result showed that the miR-7450 agomir decreased *PDE10A* mRNA expression (*p* = 0.006), but increased in the miR-7450 antagomir-transfected SCs (*p* = 0.001). The plasma treatment did not change *PDE10A* mRNA expression in SCs compared to that in the miR-7450 agomir-transfected group.

## Discussion

Short exposure to non-thermal plasma (30 s) at 11.7 kV increased immature chicken SC viability and growth, whereas prolonged plasma exposure or intensity inhibited cell viability and induced apoptosis of SCs. In a previous study, we found that inappropriate non-thermal DBD plasma treatment caused chicken embryo death in a dose-dependent manner, while optimized plasma treatment promoted embryonic development during the early stages of incubation^5^. In addition, non-thermal plasma affects sperm quality in roosters in a time- and dose-dependent manner, and optimal exposure conditions with respect of increased sperm quality have been established^6^. Immature SCs play an important physiological role in the testes for supporting germ cell development and regulating spermatogenesis^8^. *In vitro* experiments have revealed that low-dose plasma treatments enhance cell proliferation^17,18^ but that high doses induce apoptotic effects^16,19,21^ in many types of normal and cancer cells. Moreover, prolonged plasma exposure increase cell apoptosis^47^, decrease cell viability^48^, and cause cell toxicity^17^. These previous studies have suggested that inappropriate plasma treatment can affect SC viability and induce time- and dose-dependent apoptosis in chicken SCs cultured *in vitro*. Thus, the exposure condition of plasma should be sufficiently optimized for its application on SC growth *in vitro* and the possible *in vivo* application in male chickens.

The diffusion and delivery of plasma-generated ROS or stimulation of intracellular ROS-generating mechanisms as a result of non-thermal DBD plasma treatment^26,49^ has been suggested to regulate cell biological function^17^. Excessive ROS accumulation damages cellular proteins and DNA, resulting in reduced cell viability, proliferation and differentiation^24,48^, and even in cell apoptosis^26^. Our results showed that non-thermal plasma exposure excessively increases intracellular ROS levels and significantly increases MDA activity in SCs. These effects are regulated via the reduction of cellular antioxidant enzymes^21,49^ but the increase of mRNA expression of *NOX4*, which mediates the mitochondrial dysfunction, inducing ROS release^50^. NRF2 controls the homeostasis of ROS by promoting detoxification of superoxide and peroxides through SOD, PRDX, GPx, and CAT^51–53^. NRF2 is suppressed under a basal condition through binding to KEAP1, which in turn facilitates the ubiquitination and subsequent proteolysis of NRF2^54^. Our results show that plasma exposure improved the KEAP1 level but reduced the NRF2 level and mRNA expression and activity of antioxidant enzymes (SOD, CAT, GPx, and PRDX) in SCs. These findings indicate the formation of excessive ROS and the impaired antioxidant defense through disruption of the NRF2-KEAP1 signaling pathway, result in the plasma-mediated apoptosis of chicken SCs.

Mitochondria are primarily affected early in the apoptotic process and act as central coordinators of cell death^55^. Cell growth has been found to be positively correlated with mitochondrial respiratory enzyme activity and ATP production^56^. The levels of intracellular ROS increase as a byproduct of the mitochondrial respiratory chain^57^. Kaushik *et al*. showed that plasma-generated ROS activate mitochondrial-mediated apoptosis by decreasing metabolic viability, intracellular ATP level, and mitochondrial membrane potentials^58^. Plasma exposure induces the mitochondrial membrane potential reduction, mitochondrial enzymatic dysfunction and morphological changes^59^, which are resulted from the DNA oxidative damage and the disruption of DNA transcription and ATP release in mitochondria induced by the mitochondrial ROS production^58^. The findings here showed that non-thermal plasma treatment inhibits mitochondrial respiratory enzyme activity and increases JC-1 green fluorescence intensity via a low mitochondrial membrane potential, confirming induced SC apoptosis via the ROS-mediated mitochondria-dependent death pathway. Moreover, plasma exposure of SCs was found to inhibit ATP production via the downregulation of mRNA expression of *ATP* synthase subunits and ATP5A protein level. However, the ATP level in the present study contrast those with obtained in another study where plasma increases the ATP secretion and induces fluctuations in ATP level that are regarded as the representative secreted damage-associated molecular patterns in carcinoma cells^26^.

Cellular energy level can be mediated by the energy-sensing and regulatory pathway of AMPK-mTOR^35^. AMPK is involved in the renewal of cellular mitochondrial content by activating the biogenesis of new mitochondria, resulting in the inhibition of respiratory chain enzymes, which in turn decreases the level of intracellular ATP^60^. Specific deletion of AMPKα in mouse SCs leads to dysregulated energy metabolism with altered ATP production^61^. mTOR promotes cellular ATP production and mitochondrial metabolism and biogenesis^62^. In the present study, plasma exposure of chicken SCs increased *AMPK*α mRNA level and activated AMPKα phosphorylation via the downregulation of miR-7450 expression (as miRNAs induce mRNA degradation and therefore translation repression in the post-transcriptional regulation of gene expression^29,30^), whereas it reduced *mTOR* mRNA level and mTOR phosphorylation. AMPK activation suppresses the mTOR signaling pathway, further regulating cell growth and even inducing cell apoptosis^33–35^. The AMPK-mTOR signaling pathway has been reported to be involved in the disruption of SC polarity^37^, autophagy, and apoptosis in SCs under conditions of stress and mitochondrial dysfunction^38,39^. These findings reveal that plasma-downregulated miR-7450 expression may be involved in chicken SC apoptosis because of reduced mitochondrial metabolism and ATP production via regulation of the AMPK-mTOR signaling pathway.

miRNAs act as important regulators in SC proliferation, maturation, and apoptotic mechanisms via the modulation of transcription factors^12,31^ and therefore also play a crucial role in spermatogenesis. Significant changes in the expression of eight miRNAs involved in both azoospermia and non-obstructive azoospermia were found to be probably due to cytotoxicity and junction injury in SCs^63^. SC-specific deletion of dicer leads to male infertility and progressive testicular degeneration due to the absence of mature spermatozoa^32^. Transgenic mice overexpressing miR-471 in SCs suffer from compromised SC-SC adhesion at the blood–testis barrier, increased germ cell apoptosis, and impaired fertility *in vivo*^64^. A recent *in vitro* study has shown that an miR-1285 inhibitor directly upregulates AMPK expression via a 3′-UTR target site, which correlates with decreased levels of ATP and mTOR phosphorylation; this inhibited the cell viability and proliferation of immature boar SCs^12^. In the present study, we found that the miR-7450 antagomir decreased mitochondrial membrane potential, respiratory enzyme activity, ATP level, and mTOR phosphorylation through the targeting of AMPKα activation, which resulted in significant SC death. However, the miR-7450 agomir produced opposite effects on these parameters and ameliorated plasma-induced apoptotic effects. These findings help elucidate the role of miR-7450 in the regulation of non-thermal plasma-mediated SC apoptosis via AMPKα activation and the further inhibition of the mTOR signaling pathway.

Taken together, the exposure condition of non-thermal plasma should be sufficiently optimized when used for chicken SC growth *in vitro*, based on the fact that inappropriate plasma treatment induces SC apoptosis, which possibly results from the excess production of ROS via the suppression of antioxidant defense systems and the decrease of cellular energy metabolism through the inhibition of ATP release and respiratory enzyme activity in the mitochondria, a process mediated by the involvement of miR-7450 through targeting on the activation of AMPK signaling pathway. These findings provide a reference for the condition optimization of plasma technique and the potential mechanisms prior to its *in vivo* application for controlling the SC number and sperm production of cocks.

## Materials and Methods

### Culture of chicken SCs

Testes were obtained under sterile conditions from immature male chickens (Korean native chicken, approximately 30 days old) raised on a chicken farm (Jeju National University, Jeju, Republic of Korea). Animal handling protocols were approved by the Institutional Committee for Ethics in Animal Experiments of Jeju National University and all experiments were performed in accordance with the institution guidelines. SCs were isolated from the testes, evaluated for purity, and cultured using previously described methods^7^. They were counted and seeded to an appropriate density (2 × 10^5^ cells/ml) in Dulbecco’s modified Eagle’s medium/nutrient mixture F-12 (1:1) supplemented with antibiotics and 5% fetal bovine serum (Gibco, Thermo Fisher Scientific, Waltham, MA) at 37 °C in a humidified atmosphere containing 5% CO_2_. After the first 12 h, residual germ cells were removed as previously described^7^. SCs were cultured for approximately 48 h to 70–80% confluence prior to use in experiments.

### Non-thermal plasma treatment

A cell culture plate or dish was placed on the glass dielectric barrier of the plasma reactor. This allowed SCs to be directly exposed to non-thermal plasma propagating from the tip of electrode needles at varying durations and potentials following our previously described method^4,5^. Briefly, the non-thermal dielectric barrier discharge plasma reactor had two disk-shaped electrodes (100 mm) and a glass dielectric barrier (5 mm) (Fig. 1A). Sixteen needles (thickness: 1 mm; length: 2.5 mm) were evenly distributed on the surface of upper electrode. The distance gap from the needle tip to the surface of culture medium was 20 mm. Operating frequency of high voltage alternating current was 60 Hz. Argon flow rate was 2 l/min. The voltage was measured using a high voltage probe (1000×, P6015, Tektronix, Beaverton, OR, USA) and recorded by a digital oscilloscope (TBS1064, Tektronix). The discharge powers dissipated in the plasma reactor at different applied voltages were determined using a voltage-charge Lissajous plot. The applied voltage (11.7 to 27.6 kV) is corresponding to the exponentially increase of discharge power (0.05 to 14.28 W) (Fig. 1B). Plasma conditions were as follows: 11.7 kV for 30, 60, 120, and 180 s and 11.7, 16.4, 22.0, and 27.6 kV for 120 s. Afterward, plasma-treated SCs were returned to the cell incubator for 3 h before analysis. Untreated SCs were used as the control group.

### Analysis of cell viability and apoptosis

Cell viability was detected with Cell Counting Kit-8 (CCK-8; Dojindo Molecular Technologies, Kumamoto, Japan) according to the manufacturer’s instruction. Absorbance was measured at 450 nm using a GloMax Discover Multimode Detection System (Promega, Madison, WI). SC viability (%) was calculated using the following formula:

$$\begin{array}{c}({\rm{absorbance}}\,{\rm{of}}\,{\rm{treatment}}\,{\rm{group}}-{\rm{absorbance}}\,{\rm{of}}\,{\rm{blank}})/({\rm{absorbance}}\,{\rm{of}}\,{\rm{control}}\,{\rm{group}}\\ \,\,\,\,\,\,\,\,\,\,\,\,\,\,-{\rm{absorbance}}\,{\rm{of}}\,{\rm{blank}})\times 100 \% \end{array}$$

The live-cell growth status was recorded by photography using an IncuCyte Zoom System (Essen BioScience, Inc., Ann Arbor, MI).

Cell apoptosis was evaluated using annexin V/propidium iodide (PI) staining. SCs were washed with ice-cold phosphate buffered saline (PBS), centrifuged (100 × *g*) for 5 min at 4 °C, resuspended in 1× binding buffer of an Annexin V-fluorescein isothiocyanate Apoptosis Detection Kit (BD Biosciences, San Joes, CA). Five microliters of fluorescein isothiocyanate-labelled annexin V and 5 µl of PI were added to a 490 µl suspension and were gently mixed. After incubation at 25 °C for 15 min in the dark, SCs were analyzed using BD Accuri^TM^ C6 Flow Cytometer (BD Biosciences).

### Cell mitochondrial membrane potential analysis

Mitochondrial depolarization occurring in apoptosis is indicated by an increase in the green/red fluorescence intensity ratio following JC-1 dye staining. Detection of altered cell mitochondrial membrane potential was performed using a JC-1 staining kit (Sigma-Aldrich, St. Louis, MO). SCs were washed with PBS and stained with 2 µg/ml of JC-1 dye at 37 °C for 30 min in the dark. Photographs were taken using a fluorescence microscope (Olympus IX70, Tokyo, Japan) equipped with a digital camera (DP71, Olympus) at 520 nm excitation/596 nm emission. Fluorescence intensities were analyzed using ImageJ software (Rasband WS, National Institutes of Health).

### ROS production and antioxidant enzyme analysis

Intracellular ROS production was detected using DCFDA dye (Sigma-Aldrich), and MitoSOX Red (Thermo Fisher Scientific) was used for mitochondrial superoxide detection. SCs were separately stained with 15 µM DCFDA for 15 min and 5 µM MitoSOX Red for 10 min at 37 °C, followed by staining with 1 µg/ml of DAPI (Sigma-Aldrich) for 10 min. Fluorescence images of SCs were taken using a fluorescence microscope (Olympus IX70) at 492 nm excitation/520 nm emission for DCFDA, 510 nm /580 nm for MitoSOX Red, and 340 nm /488 nm for DAPI. Fluorescence intensities were analyzed using ImageJ.

SCs were assayed for MDA, SOD, CAT, and GPx levels using kits from Sigma-Aldrich and BioVision (Milpitas, CA) following the manufacturer’s instructions. Total ROS level was measured using the OxiSelect *In Vitro* ROS/RNS Assay Kit (Cell Biolabs, San Diego, CA). The fluorescence intensity of 2’,7’-dichlorodihydrofluorescein (DCF) is proportional to the total ROS level within the sample. The optical densities were measured using the GloMax Discover Multimode Detection System (Promega). Units of MDA (nmole/mg protein), SOD and CAT (U/mg protein), GPx level (mU/mg protein), and ROS (nmole DCF/mg protein) were expressed.

### Mitochondrial staining and respiratory enzyme and ATP analysis

SCs were stained using a Cell Navigator Mitochondrion Staining Kit (Green Fluorescence; AAT Bioquest, Inc., Sunnyvale, CA) at 37 °C in a 5% CO_2_ incubator for 1 h. The cells were then photographed using a fluorescence microscope at 485 nm excitation/525 nm emission. Fluorescence intensities were analyzed using ImageJ.

Mitochondria were isolated and purified from SCs using a Qproteome Mitochondria Isolation Kit (QIAGEN, Valencia, CA), according to the manufacturer’s instructions. The NADH level and enzymatic activities of cytochrome c oxidase and ATP synthase in SCs were measured using an NAD + /NADH Quantitation Colorimetric Kit (BioVision), Cytochrome Oxidase Activity Colorimetric Assay Kit (BioVision), and ATP Synthase Activity Assay Kit (Novagen, Merck KGaA, Darmstadt, Germany), according to the manufacturers’ instructions. Optical densities were measured using the GloMax Discover Multimode Detection System.

SC protein level was measured using the Bicinchoninic Acid Protein Assay Kit (Sigma-Aldrich) and adjusted to equal protein concentrations using PBS. ATP concentrations in SCs were assayed using a kit from Invitrogen (Thermo Fisher Scientific), following the manufacturer’s procedures. The light unit values of ATP level were determined using a luminometer (Sirius L Tube Luminometer, Titertek Berthold, Germany). Unit of ATP (nmole/mg protein) was expressed.

### Cell transfections

SCs were cultured in Opti-MEM^®^ reduced serum medium without antibiotics one day before transfection. At 60–80% confluence, SCs were mock-transfected (Lipofectamine^®^ RNAiMAX Regent only; Thermo Fisher Scientific) or transfected with complexes of Lipofectamine^®^ RNAiMAX, miR-7450 agomir negative control (NC; 50 nM), miR-7450 antagomir NC (100 nM), miR-7450 agomir (chemically-modified double-stranded miRNA mimic; 50 nM) in the presence or absence of non-thermal plasma treatment at 22.0 kV for 120 s, and miR-7450 antagomir (chemically-modified single-stranded miRNA inhibitor; 100 nM) from GenePharma Co., Ltd. (Shanghai, China), according to the manufacturer’s protocol. The sequence of the miR-7450 agomir was 5′- UCUGUUCUUAAGGAGGCUGAGGC-3′ and 5′-CUCAGCCUCCUUAAGAACAGAUU-3′. The sequence of the miR-7450 antagomir was 5′-GCCUCAGCCUCCUUAAGAACAGA-3′. The sequence of the miR-7450 agomir NC was 5′-UUCUCCGAACGUGUCACGUTT-3′ and 5′-ACGUGACACGUUCGGAGAATT-3′. The sequence of the miR-7450 antagomir NC was 5′-CAGUACUUUUGUGUAGUACAA-3′. Transfected SCs were incubated at 37 °C in a humidified atmosphere containing 5% CO_2_ for 6 h. The medium was replaced with fresh Opti-MEM^®^ medium, and the cells were incubated for a further 48 h.

### RT-PCR analysis

SC total RNA isolation and purification was performed using a MiniBEST Universal RNA Extraction Kit (TaKaRa Bio Inc., Kusatsu, Shiga, Japan). RT-PCR analysis for mRNA expression was performed using a PrimeScript^TM^ RT Reagent Kit (TaKaRa) and SYBR^®^ Premix Ex Taq^TM^ II (TaKaRa), according to the manufacturers’ protocols. Reverse transcription was performed at 37 °C for 15 min, and enzyme inactivation was performed at 85 °C for 5 s. The conditions used for RT-PCR were as follows: 40 cycles of initial denaturation at 95 °C for 30 s, followed by denaturation at 95 °C for 5 s and annealing for 30 s at the indicated temperatures (Supplementary Table S1).

RT-PCR analysis for miRNA expression was performed using a Mir-X^TM^ miRNA qRT-PCR SYBR^®^ Kit (TaKaRa), according to the manufacturer’s protocol. Reverse transcription was performed at 37 °C for 1 h, and enzyme inactivation was performed at 85 °C for 5 min. Ninety microliters of RNase-free dH_2_O was then added to dilute the cDNA. The following conditions were used for RT-PCR: 40 cycles of initial denaturation at 95 °C for 10 s, followed by denaturation at 95 °C for 5 s and annealing at 60 °C for 20 s. Primer sequences used for RT-PCR are shown in Supplementary Table S1.

The cycle threshold value was calculated based on triplicate measurements. The equivalent dilutions was determined according to the standard curve and then normalized against a housekeeping gene (*β-actin* for mRNA, U6 for miRNA). Relative expression levels were calculated using the 2^−ΔΔCT^ method.

### Western blotting

For extraction of total protein, SCs were scraped and resuspended in a lysis buffer (5 mM phosphate buffer, pH 7.2, 0.1% Triton X-100, 10 mM sodium fluoride, 1 mg/l chymostatin, and 1 mM phenylmethylsulfonyl fluoride) at 4 °C for 30 min prior to centrifugation for 10 min at 11000 × *g*. Protein concentration was measured using the Bicinchoninic Acid Protein Assay Kit and adjusted to equal protein concentration using lysis buffer. Western blotting was performed using protein samples extracted from three independent cell cultures for each group. SC protein was separated using a sodium dodecyl sulfate-polyacrylamide gel electrophoresis (12%) and then transferred to polyvinylidene fluoride membranes via wet electrophoretic transfer (Bio-Rad, Hercules, CA). Membranes were blocked in PBS-0.08% Tween containing 5% skim milk or 3% BSA for 2 h at room temperature and subsequently incubated with primary detection antibodies at 4 °C overnight. The following antibodies were used: anti-NRF2 (mouse monoclonal; Santa Cruz Biotechnology; 1:200), anti-KEAP1 (mouse monoclonal; Santa Cruz Biotechnology; 1:200), anti-PRDX4 (mouse monoclonal; Santa Cruz Biotechnology; 1:200), anti- ATP5A (rabbit polyclonal; Abcam; 1:250), anti-phospho-AMPKα (Thr172, p-AMPKα; rabbit polyclonal; Cell Signaling Technology; 1:1,000), anti-AMPKα (rabbit polyclonal; Cell Signaling Technology; 1:1,000), anti-phospho-mTOR (Ser2448, p-mTOR; rabbit monoclonal; Cell Signaling Technology; 1:1,000), anti-mTOR (rabbit polyclonal; Cell Signaling Technology; 1:1,000), anti-beta actin (rabbit polyclonal; Bioss; 1:1,000). Secondary antibodies were goat anti-mouse (Santa Cruz Biotechnology; 1: 5,000) and goat anti-rabbit (Abcam; 1: 5,000) immunoglobulin G conjugated to horseradish peroxidase. Membranes were rinsed three times for 5 min. After incubation with the secondary antibody for 2 h at room temperature, protein bands were visualized via SuperSignal West Pico Plus Chemiluminescent Substrate (Thermo Fisher Scientific). Intensity of bands was analyzed using ImageJ. The densitometric values of the NRF2, KEAP1, PRDX4, and ATP5A bands were normalized against the relevant β-actin. The densitometric values for p-AMPKα, AMPKα, p-mTOR, and mTOR bands were normalized against the β-actin prior to the calculation of the p-AMPKα/AMPKα and p-mTOR/mTOR ratios.

### Statistical analysis

Data are represented as the mean ± standard deviation (SD) of three independent experiments. Statistical analysis was performed using the Statistical Package for the Social Sciences (SPSS version 16.0; SPSS, Chicago, IL). Statistically significant differences among treatment groups were determined by one-way ANOVA and Fisher’s least significant difference (LSD) tests. *P*-values of < 0.05 were considered significant.

### Data availability

The datasets generated during and analysed during the current study are available from the corresponding author on reasonable request.

## Electronic supplementary material

Supplementary Information
