# Supplementary material for: MicroRNA-7450 regulates non-thermal plasma-induced chicken Sertoli cell apoptosis via adenosine monophosphate-activated protein kinase activation
Source: Sci Rep. 2018 Jun 8;8:8761. doi: 10.1038/s41598-018-27123-8 (PMC5993736; doi:10.1038/s41598-018-27123-8)
Supplement: Supplementary file 1 — Supplementary Information [file 41598_2018_27123_MOESM1_ESM.pdf]

Supplementary Information

MicroRNA-7450 regulates non-thermal plasma-induced chicken Sertoli cell apoptosis via adenosine monophosphate-activated protein kinase activation

Jiao Jiao Zhang[1], Xian Zhong Wang[2], Huynh Luong Do[1], Nisansala Chandimali[1], Tae Yoon Kang[1], Nameun Kim[1], Mrinmoy Ghosh[1], Sang Baek Lee[3], Young Sun Mok[3], Seong Bong Kim[4], Taeho Kwon[5, *], Dong Kee Jeong[1, 5, *]

[1] Laboratory of Animal Genetic Engineering and Stem Cell Biology, Department of Advanced Convergence Technology and Science, Jeju National University, Jeju 63243, Republic of Korea

[2] Chongqing Key Laboratory of Forage and Herbivore, College of Animal Science and Technology, Southwest University, Chongqing 400715, P. R. China

[3] Department of Chemical and Biological Engineering, Jeju National University, Jeju 63243, Republic of Korea

[4] Plasma Technology Research Center, National Fusion Research Institute, Gunsan-si, Jeollabuk-Do 54004, Republic of Korea

[5] Laboratory of Animal Genetic Engineering and Stem Cell Biology, Subtropical/Tropical Organism Gene Bank, Jeju National University, Jeju 63243, Republic of Korea

[*]Correspondence and requests for materials should be addressed to D.K.J. (E-mail: ngejeong@gmail.com) and T.K. (E-mail: taehokwonk@gmail.com)

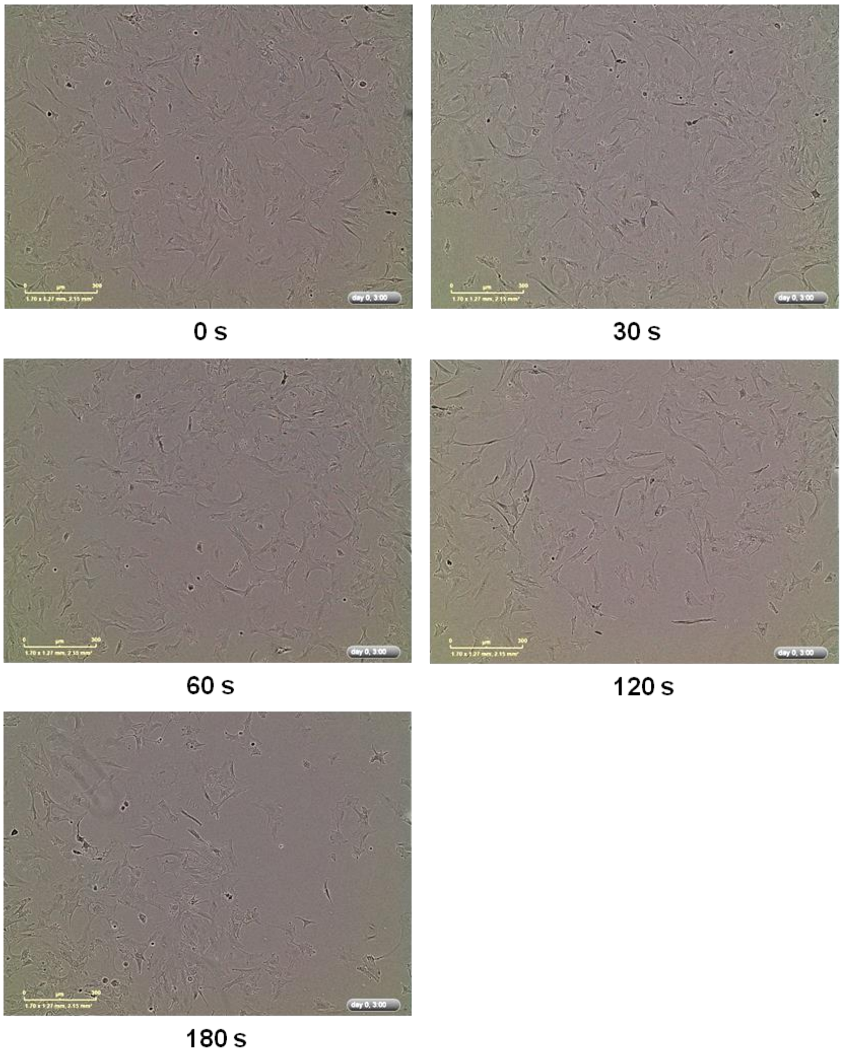

Supplementary Figure S1. Growth status of SCs exposed to 11.7 kV for different durations. Scale bar: 300 μm.

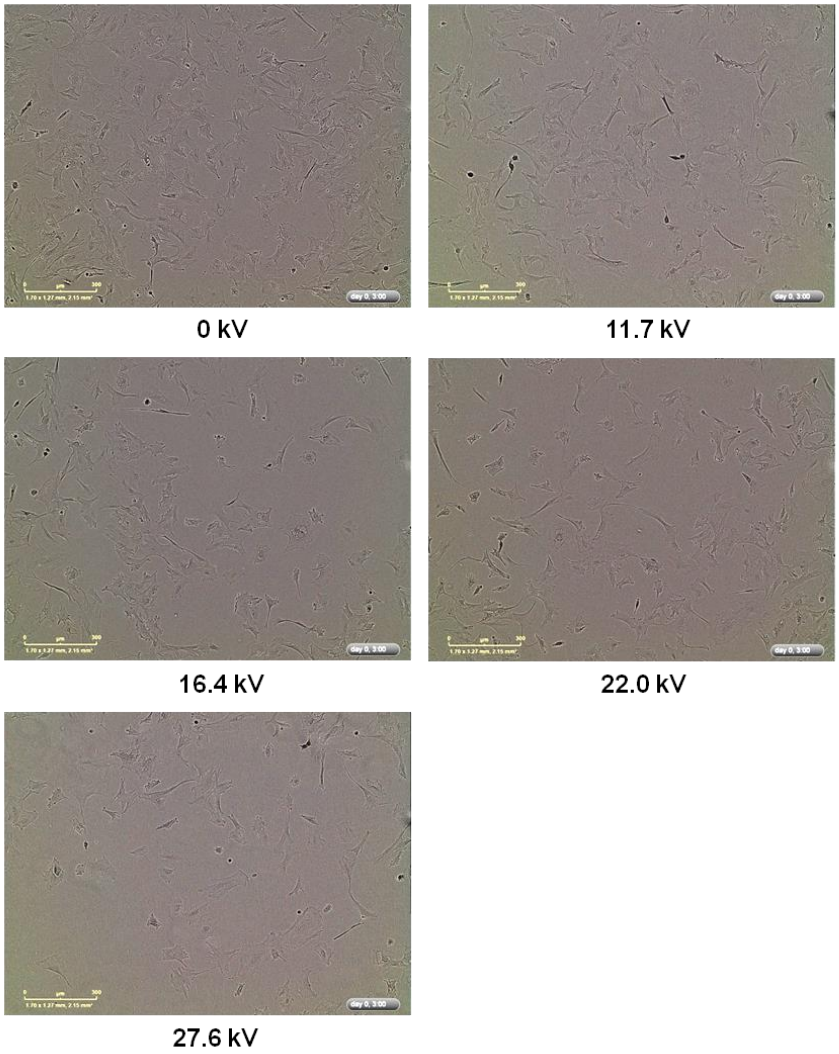

Supplementary Figure S2. Growth status of SCs exposed to different potentials for 120 s. Scale bar: 300 μm.

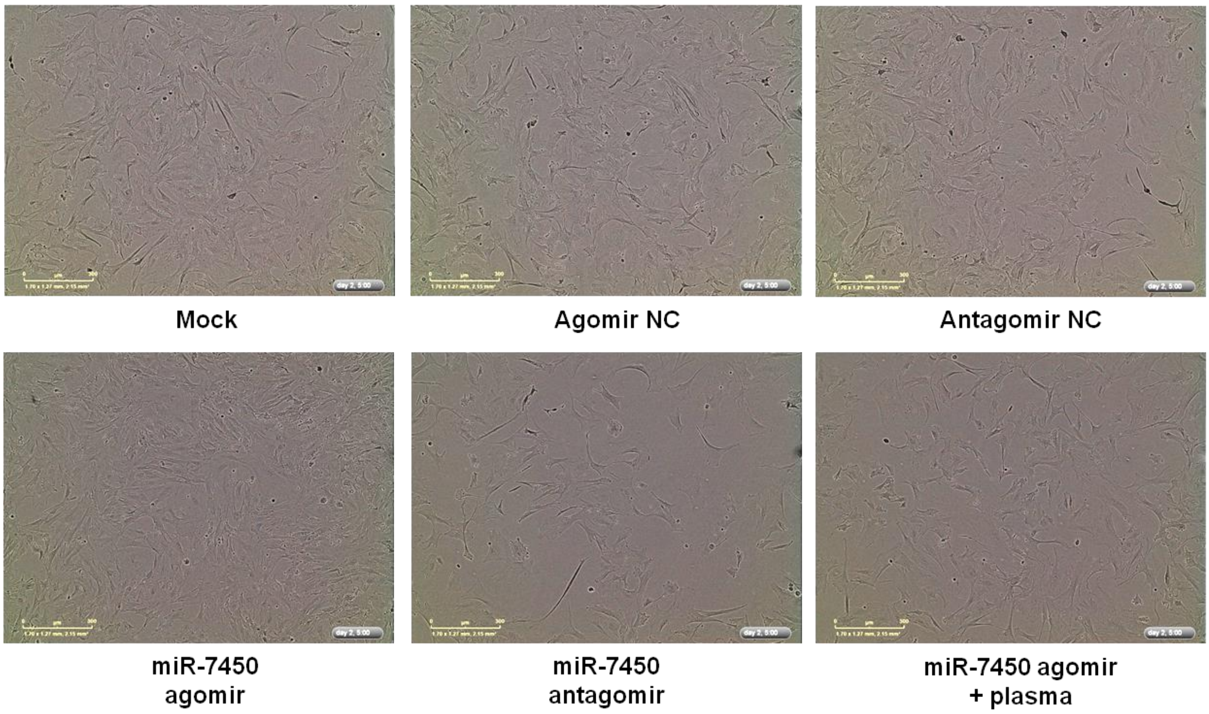

Supplementary Figure S3. Growth status of SCs transfected with miR-7450 agomir and antagomir, and miR-7450 agomir-transfected group treated with 22.0 kV of plasma for 120 s. Scale bar: 300 μm.

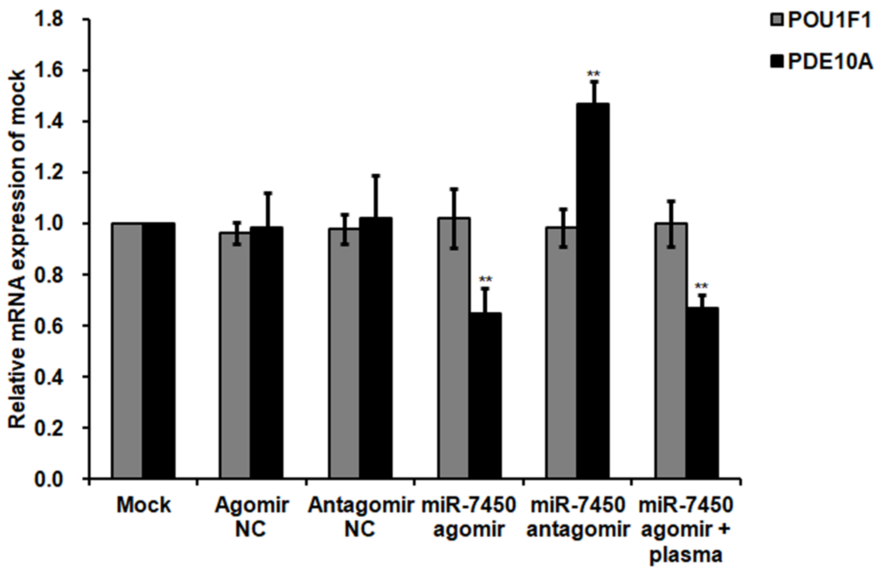

Supplementary Figure S4. RT-PCR analysis of a non-target gene (*POU1F1*) and an unrelated target gene (*PDE10A*) of miR-7450 in SCs transfected with miR-7450 agomir and antagomir, and miR-7450 agomir-transfected group treated with 22.0 kV of plasma for 120 s. Data are represented as the mean ± SD (n=3 per group). *, $p < 0.05$; **, $p < 0.01$, according to one-way ANOVA and LSD test.

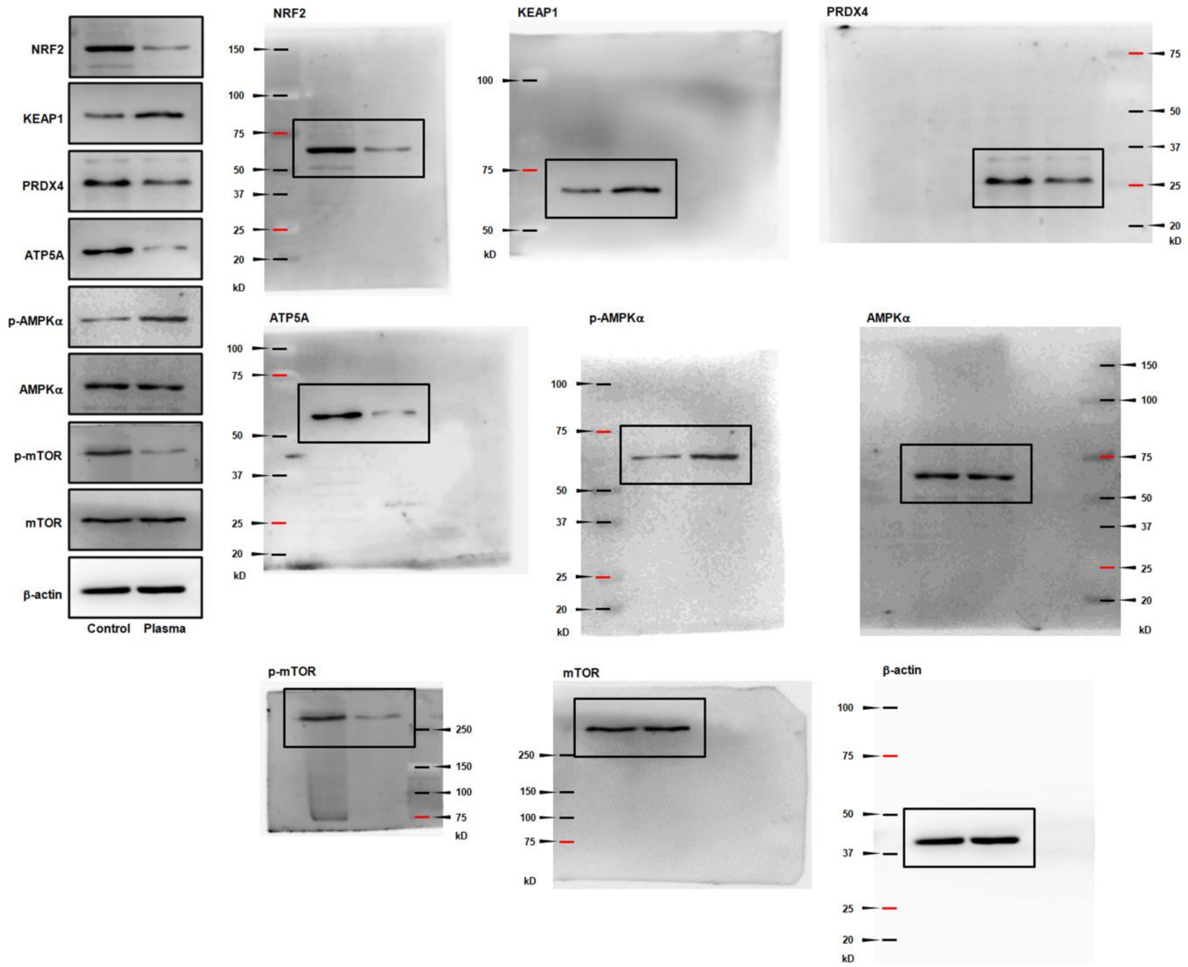

Supplementary Figure S5. Western blot analysis of the protein levels of NRF2, KEAP1, PRDX4, ATP5A, p-AMPKα, AMPKα, p-mTOR, and mTOR in SCs exposed to 22.0 kV of plasma for 120 s. Uncropped immunoblot scans for Fig. 3A. The grouping of gels/blots cropped from different gels. All blots were visualized with 5 min exposure time.

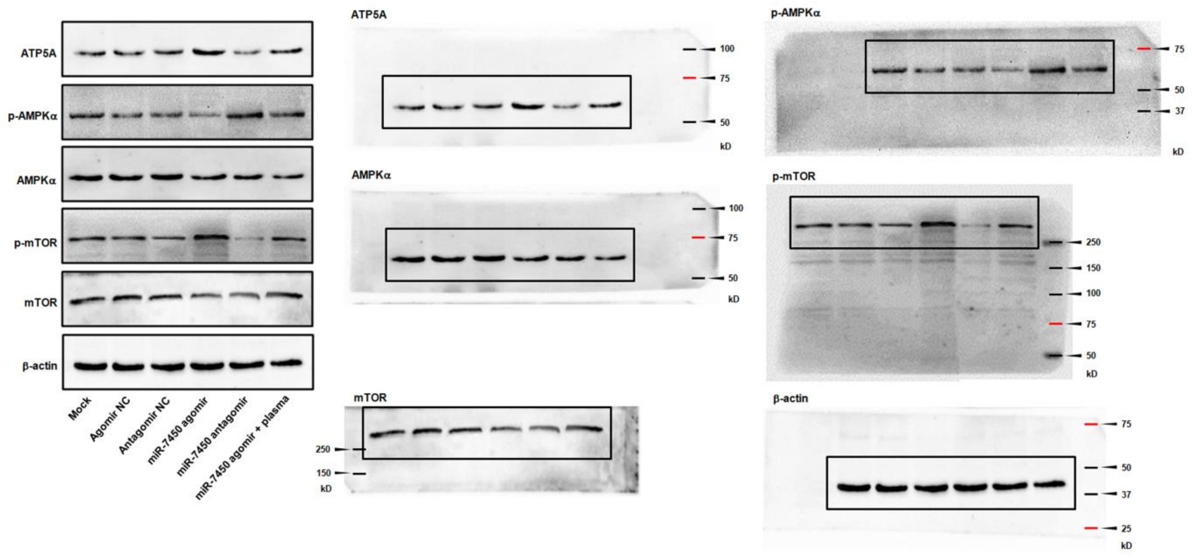

Supplementary Figure S6. Western blot analysis of the protein levels of ATP5A, p-AMPKα, AMPKα, p-mTOR, and mTOR in SCs trasfected with miR-7450 agomir and antagomir, and miR-7450 agomir-transfected group treated with 22.0 kV of plasma for 120 s.

Uncropped immunoblot scans for Fig. 3F. The grouping of gels/blots cropped from different gels. All blots were visualized with 5 min exposure time.

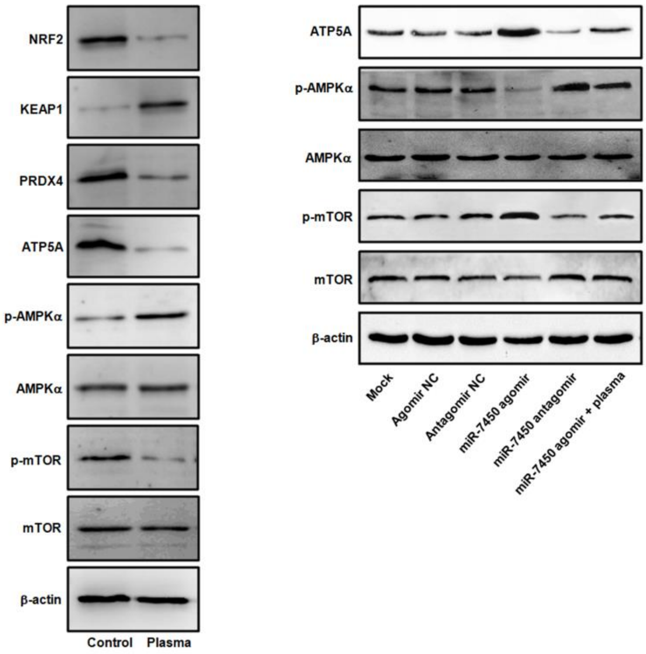

Supplementary Figure S7. One independent replicate on chicken SC protein expression. (**A**) Western blot analysis of protein bands in SCs exposed to 22.0 kV of plasma for 120 s. (**B**) Western blot analysis of protein bands in SCs trasfected with miR-7450 agomir and antagomir, and miR-7450 agomir-transfected group treated with 22.0 kV of plasma for 120 s.

Supplementary Table S1. Primer sequences for the RT-PCR

**Gene**	**Sequence number**	**Sequence position**	**Product length (bp)**	**Annealing Temperature (°C)**	**Sequence (5'to3')**
β-actin	NM_205518.1	625-818	194	57	F: GTGCGTGACATCAAGGAGAAGC<br>R: CCACAGGACTCCATACCCAAGA
NOX4	NM_001101829.1	28-157	130	57	F: CGAGGATCTCAGAAGGTTGC<br>R: GAGCATTCACCAGATGAGCA
NRF2	NM_205117.1	484-619	136	57	F: AAAACGCTGAACCACCAATC<br>R: GCTGGAGAAGCCTCATTGTC
KEAP1	KU321503.1	1227-1485	259	57	F: GTATCACAGCAGCGTGGAGA<br>R: GGCGTAGATGCAGTTGTTGA
SOD	NM_205064.1	106-278	173	55	F: ATTACCGGCTTGTCTGATGG<br>R: CCTCCCTTTGCAGTCACATT
CAT	NM_001031215.2	1067-1276	210	55	F: CTCATTCCAGTGGGCAAGAT<br>R: GTAGGGGCAATTCACAGGAA
GPx	NM_001277853.2	353-474	122	55	F: ATGTTCGAGAAGTGCGAGGT<br>R: ATGATGTACTGCGGGTTGGT
PRDX4	XM_001233999.3	595-733	139	56	F: TGCACTTAGGGGCCTTTTCA<br>R: TTCTCCATGCTTGTCCGTGT
ATP5A1	NM_204286.1	1207-1364	158	57	F: GGTATCCGTCCAGCCATCAA<br>R: GCATCCAAATCAGACCCAAACT
AMPKα	NM_001039605.1	726-943	218	57	F: GGAGGCGTGTTTTACATCCC<br>R: AACTTCTCACAGACCTCCCG
mTOR	XM_417614.5	119-309	191	57	F: TGAAGGGGTCAAGGCAATCC<br>R: GGCGAGCAGTGGTTGTGGAT
POU1F1	NM_204319.1	560-754	195	57	F: ATGTTGGCGAAGCACTGGC<br>R: GCTTCCTCTTCCGCTCATTCA
PDE10A	XM_004935551.2	3258-3412	155	57	F: CCACATCAACGATGGAACAG<br>R: ATACAGGGCAAGGTCTGTGG
U6	NR_003027.2	66-85	20	60	F: CGCAAGGATGACACGCAAAT
miR-7450	MI0024118	1-20	20	60	F: TCTGTTCTTAAGGAGGCTGA
